# Phenotypic Detection of Clonotypic B Cells in Multiple Myeloma by Specific Immunoglobulin Ligands Reveals their Rarity in Multiple Myeloma

**DOI:** 10.1371/journal.pone.0031998

**Published:** 2012-02-22

**Authors:** Martin Trepel, Victoria Martens, Christian Doll, Janina Rahlff, Barbara Gösch, Sonja Loges, Mascha Binder

**Affiliations:** 1 Department of Oncology and Hematology, BMT with section Pneumology, University Medical Center Hamburg-Eppendorf, Hubertus Wald Tumorzentrum/University Cancer Center Hamburg, Hamburg, Germany; 2 Department of Oncology and Hematology, University Medical Center Freiburg, Freiburg, Germany; Carl-Gustav Carus Technical University-Dresden, Germany

## Abstract

In multiple myeloma, circulating “clonotypic” B cells, that express the immunoglobulin rearrangement of the malignant plasma cell clone, can be indirectly detected by PCR. Their role as potential “feeder” cells for the malignant plasma cell pool remains controversial. Here we established for the first time an approach that allows direct tracking of such clonotypic cells by labeling with patient-specific immunoglobulin ligands in 15 patients with myeloma. Fifty percent of patients showed evidence of clonotypic B cells in blood or bone marrow by PCR. Epitope-mimicking peptides from random libraries were selected on each patient's individual immunoglobulin and used as ligands to trace cells expressing the idiotypic immunoglobulin on their surface. We established a flow cytometry and immunofluorescence protocol to track clonotypic B cells and validated it in two independent monoclonal B cell systems. Using this method, we found clonotypic B cells in only one out of 15 myeloma patients. In view of the assay's validated sensitivity level of 10^−3^, this surprising data suggests that the abundance of such cells has been vastly overestimated in the past and that they apparently represent a very rare population in myeloma. Our novel tracing approach may open perspectives to isolate and analyze clonotypic B cells and determine their role in myeloma pathobiology.

## Introduction

Multiple myeloma is the second most common hematological malignancy worldwide [1], [2]. The disease is characterized by a monoclonal expansion of malignant plasma cells (PC) in the bone marrow, committed to the production of a patient-specific monoclonal serum immunoglobulin, the “paraprotein”. While many patients initially respond well to PC-directed therapies, almost all patients relapse and ultimately succumb to the disease [3]. Although the malignant PC clone is held responsible for most myeloma-related morbidity such as anemia, renal failure and bone lesions, it has been a subject of debate whether or not these cells possess enough proliferative potential to sustain the disease. The discovery of (surface) immunoglobulin-positive B cells expressing the same patient-individual variable region immunoglobulin (Ig) rearrangement as the malignant PC clone – so called clonotypic B cells – has therefore fueled speculations about a potential (pre-) malignant B cell compartment with stem cell like properties feeding the malignant PC compartment in multiple myeloma [4], [5], [6], [7], [8]. Attempts have been made to use anti-idiotypic antibodies in the detection of B cell populations with clonotypic surface Ig, but their specificity has been limited, since they react with more than one myeloma Ig and also recognize a number of normal B cell clones [9], [10]. Molecular detection of potential myeloma precursor B cells has therefore been mainly based on PCR amplification of the patient-specific Ig rearrangement from either peripheral blood or bone marrow-derived DNA or cDNA (mRNA) [11], [12], [13], [14], [15], [16], [17]. To discriminate between contaminating IgG- or IgA-positive PCs and clonotypic B cells, many studies worked on the purified CD19-positive B cell fraction and/or used IgM-specific primers for immunoglobulin gene amplification (although some studies looked at other isotypes as well). The percentage of patients in which myeloma-related Ig-positive clonotypic B cells could be detected ranged between 40% and 87% [15], [17], [18]. Limiting dilution PCRs indicated that in myeloma patients between 0.24% and 25% of peripheral blood mononuclear cells (PBMC) and up to 66% of all peripheral B cells represent clonotypic B cells [15], [16], [17]. This data implies that a substantial proportion of B cells are clonally related to the malignant PC clone. However, a rather high interpatient and interstudy variability (with some studies suggesting lower percentages of clonotypic B cells [18], [19], [20]), possibly related to methodical heterogeneity of the PCR technique, as well as potential confounders (unspecific primer annealing or expression of atypical, non-clinical Ig transcripts by myeloma subclones [21]) may limit the validity of such PCR-based approaches. When looking at different stages of the disease, the estimated frequencies of such clonotypic cells varied between lower levels after chemotherapy and higher levels in relapsed disease [14], [15], [16], [22], [23], [24], [25]. This data has been interpreted as preliminary evidence that these cells do not merely represent non-malignant clonotypic remnant B cells which had initially produced the malignant plasma cell clone, but supports their role as an active “feeder” in myelomagenesis and myeloma progression. To directly and functionally address the biological significance of this B cell population, peripheral B cells from myeloma patients have been xenotransplanted into immunodeficient mice. These studies produced inconsistent findings [26], [27], [28], [29] [30], [31]. Importantly, if the hypothesis of an active B cell “feeder” population holds true, one would expect these B cells to mirror many genetic changes observed in the malignant PC compartment. Towards this end, several studies have compared the genetic lesions in PCs with those in the peripheral B cell compartment, supporting a model of intraclonal evolution at the PC level [32], [33], [34], [35], [36], [37], [38]. More evidence questioning the role of clonotypic B cells as “feeders” of the malignant PC compartment came from a recent study on the clonal hierarchy in light-chain myeloma [38] and from the analysis of class switch recombination from IgM to IgG or IgA in multiple myeloma [39].

Taken together, the concept of (pre-)malignant surface Ig-positive clonotypic B cells feeding the malignant PC compartment in a stem cell like manner is still highly controversial. One of the major problems hampering scientific progress can be ascribed to the fact that clonotypic B cells have been only detectable by PCR so far. Besides general concerns regarding this approach as indicated above, a major drawback of PCR-based methods is that the cells of interest cannot be morphologically identified or even directly isolated for further analyses to determine their biological role.

To overcome this critical challenge irrespective of the controversy on the functional relevance of conotypic cells in myeloma, we set out to develop a tool to specifically detect and isolate these cells for exact quantification and to obtain the possibility of their further functional characterization in future studies. Therefore, we established patient-individual ligands mimicking the epitope recognized by the myeloma immunoglobulin to specifically target clonotypic surface Ig-positive B cells from myeloma patients.. Our analysis of 15 myeloma patients indicates, that clonotypic B cells are a very rare event in myeloma and that their absolute numbers and relative frequencies have been significantly overestimated by indirect PCR-based quantification. This study, as a matter of fact, does not provide evidence to reject the “feeder” B cell hypothesis. However, the limited percentage of patients with PCR-detectable and ligand-detectable clonotypic B cells challenges the concept of an active pre-switch B cell “feeder” compartment as a general principle and indispensable prerequisite for myeloma pathogenesis and progression.

## Materials and Methods

### Ethics statement

Blood samples of myeloma patients visiting the Freiburg and Hamburg Medical Centers' outpatient units were obtained after informed written consent. This study was specifically approved by the institutional review board of the University of Freiburg and the Physician's Association in Hamburg. Control cells were obtained from healthy donors (HD). Residual bone marrow obtained for routine clinical testing was banked after completion of diagnostic procedures and used for this study after informed consent.

### Patients' characteristics

A total of 15 myeloma patients were included in this study. Table 1 summarizes the clinical characteristics, including first diagnosis, treatment and remission status at the time of sample collection.

**Table 1:** Clinical characteristics of myeloma patients.*

Patient Code	Diagnosis	Date of clinical sample acquisition	Treatment	Remission status (at date of sample acquisition)
MM001	06/2003	06/2003 and 07/2008	autologous stem cell transplant (11/2004), idiotype vaccination (06-11/2005), lenalidomide maintenance (06/2006-02/2007), lenalidomide/dexamethasone (03/2008-07/2008)	PR/SD
MM003	02/2002	02/2002	autologous stem cell transplant (05/2003), thalidomide maintenance, bortezomib (02-04/2005), idiotype vaccination (06-11/2005), cyclophosphamide/bortezomib/dexamethasone (02-07/2006)	active disease
MM008	03/2005	03/2005	autologous stem cell transplant (11/2005), allogeneic stem cell transplant (02/2006)	active disease
MM020	05/2005	05/2005 and 09/2008	autologous stem cell transplant (11/2005), idiotype vaccination (08/2006-09/2008)	PR
MM021	09/2000	05/2009	autologous stem cell transplant (11/2001 and 07/2007), lenalidomide/dexamethasone (07-12/2008), bortezomib/dexamethasone (05/2009)	PD
MM022	05/2004	05/2009	autologous stem cell transplant (12/2005), bortezomib/dexamethasone (12/2006-08/2007), lenalidomide/dexamethasone (01-09/2008), thalidomide/bortezomib/dexamethasone (10/2008-02/2009), allogeneic stem cell transplant (02/2009)	PD
MM023	12/2008	03/2009	bortezomib/dexamethasone (01-03/2009)	PR
MM025	09/2005	08/2009	autologous stem cell transplant (11/2005 and 01/2006), bortezomib/dexamethasone (07-08/2009)	PD
MM026	08/2009	08/2009	-	active disease
MM031	11/2007	04/2010	bortezomib/dexamethasone (12/2007-01/2008), autologous stem cell transplant (05/2008 and 08/2008), bortezomib maintenance	PD
MM032	01/2011	01/2011	autologous stem cell transplant (06/2011 and 09/2011)	active disease
MM034	02/2011	02/2011	-	active disease
MM036	2007	02/2011	bortezomib (01/2011)	PR
MM048	03/2011	03/2011	autologous stem cell transplant (09/2011)	active disease
MM050	01/2003	04/2011	intermittent lenalidomide (01/2003-04/2011), bortezomib/dexamethasone (04-08/2011)	PD

*MM = multiple myeloma, PR = partial remission, SD = stable disease, PD = progressive disease.

### Preparation of clinical samples and immunoglobulin purification

Peripheral blood mononuclear cells (PBMC) were isolated from the blood of myeloma patients by Ficoll density gradient centrifugation (Biacoll, Biochrom AG, Berlin, Germany). Viable PBMCs were resuspended in 90% fetal calf serum (FCS) and 10% dimethyl sulfoxide (DMSO) and stored in liquid nitrogen until use for subsequent FACS analysis, RNA preparation (Roboklon Kit, Roboklon, Berlin, Germany) and cDNA generation (Omniscript^(R)^ RT Kit by Qiagen, Hilden, Germany). The remaining serum was used for Ig purification by protein-A sepharose (GE Healthcare, Buckinghamshire, UK) for IgG paraproteins and Jacalin (Pierce, Rockford, Illinois) for IgA paraproteins as described by the supplier. Bone marrow samples collected from myeloma patients were treated with a lysis buffer (155 mM ammonium chloride, 10 mM potassium hydrogen carbonate and 1 mM ethylenediaminetetraacetic acid) to eliminate erythrocytes. The bone marrow mononuclear cells (BMMNCs) were resuspended in 90% FCS and 10% DMSO, stored in liquid nitrogen and used for the same applications as described for PBMCs. The Burkitt's lymphoma cell line CA46 (ATCC #CRL-1648**™**) and PBMCs from a CLL patient (CLL024), isolated by Ficoll density gradient centrifugation and containing more than 90% monoclonal B cells, were included as controls.

### Sequencing of immunoglobulin variable regions of malignant plasma cell clones

To amplify Ig variable heavy and light chain genes from bone marrow, cDNA of myeloma patients, polymerase chain reactions (PCR) were performed with family-specific degenerated leader primers and the corresponding reverse primer either designed for the kappa or lambda light chain constant region or for the IgG or IgA heavy chain constant region (Table S1). PCR products of correct size were excised from agarose gels, purified (HiYield^(R)^ PCR Clean-up/Gel Extraction Kit by SLG, Gauting, Germany) and subcloned into pJet1.2 cloning vector (Fermentas, St. Leon-Rot, Germany) according to the manufacturer's instructions. A minimum of eight clones were sequenced per variable region (Seqlab, Göttingen, Germany).

### Detection of IgM-positive clonotypic B cells by semi-nested PCR

**Figure 1:**
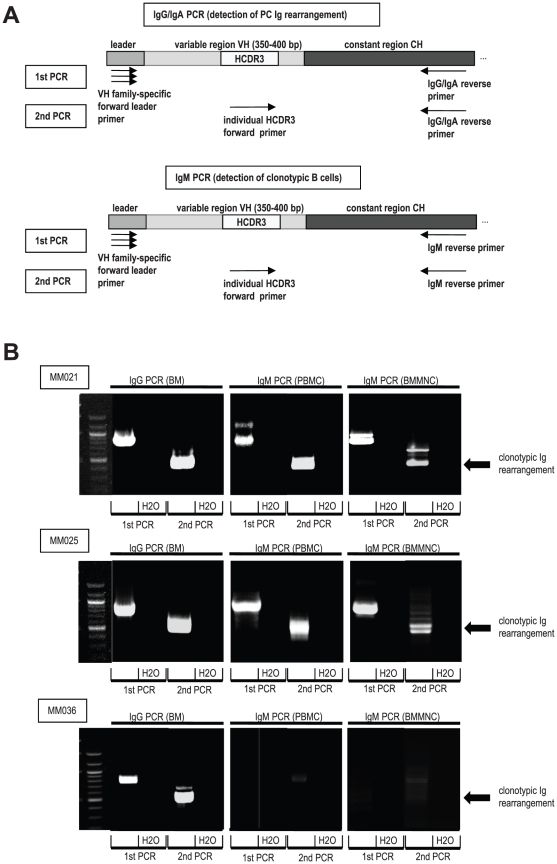
Detection of clonotypic Ig rearrangements by semi-nested PCR. **A:** Illustration of semi-nested PCR approach for the detection of clonotypic Ig rearrangements. Ig genes were amplified using VH family-specific forward leader primers and constant region specific reverse primers (1^st^ PCR). A secondary PCR was used to specifically amplify the clonotypic Ig rearrangement with a patient-individual HCDR3-specific primer. PC = plasma cell, Ig = immunoglobulin, VH = heavy chain variable region, CH = heavy chain constant region, HCDR3 = heavy chain complementarity determining region 3. **B:** Detection of clonotypic Ig-rearrangements in myeloma PBMCs and BMMNCs of patients MM021, MM025 and MM036. Semi-nested PCRs were performed as described in A. PCR products were loaded on agarose gels stained with ethidium bromide. MM = Multiple Myeloma, PBMC = peripheral blood mononuclear cell, BMMNC = bone marrow mononuclear cell.

For detection of IgM-positive clonotypic B cells from peripheral blood and bone marrow, a semi-nested PCR approach was established as illustrated in Figure 1A. Therefore, unless indicated otherwise, patient-specific heavy chain complementarity determining region 3 (HCDR3) forward primers were designed (Table S1). As a positive control and in order to test the individual HCDR3 primers, the PC's Ig rearrangement was amplified by semi-nested PCR from BMMNC cDNA (1^st^ PCR: family-specific forward primer + IgG (or IgA, respectively) reverse primer, 2^nd^ PCR: HCDR3 forward primer+IgG (or IgA, respectively) reverse primer; Figure 1A, upper panel, and Figure 1B, left panels). To detect IgM-positive clonotypic B cells from PBMC- or BMMNC-derived cDNA, the IgG/IgA reverse primer was replaced by an IgM reverse primer (Figure 1A, lower panel, and Figure 1B middle and right panels). PCR products were electrophoresed in agarose gels and visualized by ethidium bromide staining.

### Recombinant expression of myeloma Ig

cDNAs from primary myeloma cells of patients MM001, MM003, MM008 and MM020 were prepared and Fab fragments containing the variable heavy and light chain regions of the respective myeloma Ig were produced commercially (CellGenix, Freiburg, Germany) as described previously [40], [41]. Fab fragments were purified by His-tag affinity chromatography. The Ig of myeloma patient MM021, CLL patient 024 and of the Burkitt's lymphoma cell line CA46 was cloned into the plasmid pBUD, expressed as IgG1 antibody in HEK293T cells and purified by protein-A affinity chromatography [42].

### Random phage display peptide libraries

Four random phage display peptide libraries were used: three linear (X7, X12, X18) and one beta-sheet conformation (X4CX6CX4) (C = cysteine; X = random amino acid). The X12 library was purchased from New England Biolabs (Frankfurt, Germany) and processed as recommended by the manufacturer. All other libraries were generated as described previously [43], [44], [45], [46], [47], [48], [49].

### Phage library selection

The libraries were screened on recombinantly expressed myeloma Ig or purified paraprotein as previously described [50], [51] after a two-fold negative selection on polyclonal human Ig (Octapharma, Lachen, Switzerland). Random clones from the third or fourth panning round were sequenced (Seqlab, Göttingen, Germany).

### Single clone phage binding

Phage clones (10^8^ transducing units [TU]) were incubated on myeloma Ig (Fab or purified paraprotein) and control Ig. Bound phage were either quantified by ELISA as suggested by the NEB phage display manual or – more accurately - based on E. coli K91kan or ER2738 (for X12 phage) bacterial infection [43], [50], [51]. Either insertless fd tet or a phage displaying the random insert YMTPPLSSQQKS (from X12 library) were used as control phage for all experiments.

### GST-fusion protein and competition assays

The oligonucleotide encoding the phage-derived peptide FLNGCDKEDWMCWVTT was digested with BamHI and EcoRI, and cloned into pGEX-2TK (Amersham, Buckinghamshire, UK). Proteins were expressed in E. coli BL21 (Invitrogen, Camarillo, California) and purified according to the manufacturer's instructions. Competition experiments were done with GST-fusion proteins as described [50].

### Detection of surface Ig-positive clonotypic B cells by immunofluorescence using patient-individual ligands

Myeloma patients' PBMCs, the Burkitt's lymphoma cell line CA46 (ATCC #CRL-1648**™**), primary CLL cells (CLL024) and a variety of control cells (healthy donor and CLL PBMCs as well as the Burkitt's lymphoma cell line DG-75 [ATCC #CRL-2625™]) were incubated for 10 min with a 100-fold excess of the respective selected phage, washed with PBS, sedimented on adhesion slides (Marienfeld, Lauda-Königshofen, Germany), fixed with 2% paraformaldehyde and blocked with 3% BSA. Insertless fd tet and random insert phage YMTPPLSSQQKS were used as controls. Bound phage were stained with a polyclonal anti-fd bacteriophage biotin-conjugated antibody (Sigma, Saint Louis, Missouri) followed by fluorescent labeling with streptavidin-fluoresceinisothiocyanate (streptavidin-FITC, Invitrogen, Camarillo, California). Nuclei were counterstained with DAPI-containing mounting medium (VectaShield, Vector Laboratories, Burlingame, California). Images were obtained by confocal microscopy (Leika TCS SP2) and analysed by Leika Confocal Software (LCS).

### Detection of surface Ig-positive clonotypic B cells by flow cytometry using patient-individual epitope-mimicking ligands

To detect B cells expressing clonotypic Ig on their surface, myeloma patients' PBMC/BMMNC, the Burkitt's lymphoma cell line CA46 and primary CLL cells were incubated with a 100-fold excess of phage binding selectively to the respective patient's or cell line's Ig for 10 minutes on ice. Insertless fd tet and random insert phage YMTPPLSSQQKS were used as controls. After fixation (Fix&Perm Kit by ADG Bio Research GmbH, Kaumberg, Austria), cell-bound phage were detected with an anti-fd bacteriophage biotin-conjugated antibody (Sigma, Saint Louis, Missouri) followed by fluorescent labeling with streptavidin-FITC (Invitrogen, Camarillo, California). B cells were stained with a CD19 allophycocyanin-conjugated antibody (CD19-APC, Abcam, Cambridge, UK). To exclude unspecific binding, stainings were also performed on healthy donor PBMC. To establish the sensitivity level of this protocol, spiking experiments with stained and unstained CA46 and primary CLL024 cells were conducted. All measurements were taken on a FACSCalibur flow cytometer (BD, Heidelberg, Germany) and analyzed with the FlowJo software (Treestar).

## Results

### Detection of IgM-positive clonotypic B cells by PCR

15 myeloma patients with different clinical features were included in this study (Table 1). The variable heavy (VH) and variable light (VL) chain Ig rearrangement of the malignant PC clone was amplified using family- and isotype-specific primer pairs (Table S1) and sequenced as described in the Methods section. Table 2 lists the myeloma patients' Ig isotype, VH and VL gene usage and complementarity determining region 3 sequences (HCDR3 and LCDR3) as well as the mutational status of the VH and VL chain.

**Table 2:** Malignant plasma cell immunoglobulin rearrangements.*

Patient Code	Isotype	VH Gene	VL Gene	HCDR3 Sequence	LCDR3 Sequence	Mutational Status VH	Mutational Status VL
MM001	IgG κ	5-51	1-33	CVRPRIRERGPIPLDFW	CQQYETFPGSF	M (93.4%)	M (94.6%)
MM003	IgG κ	2-26	1-39	CARVSVERRRGSAFDVW	CQQSYTSPRTF	M (93.5%)	M (90.7%)
MM008	IgG κ	6-1	1-39	CARESPSGILNDYDQYYYGMDVW	CLQSHSNPNTF	M (90.6%)	M (91.8%)
MM020	IgG κ	4-39	3-20	CAGRGSNFDSDSRDFIIFDSW	CQQYAASPLTF	M (90.4%)	M (94.3%)
MM021	IgG λ	3-21	2-23	CARVQIPAALDSW	CCAFGGSVTVF	M (94.1%)	M (93.7%)
MM022	IgG κ	n.e.	n.e.	n.e.	n.e.	n.e.	n.e.
MM023	IgG κ	5-51	4-1	CACPSRYSSVWRIDYW	CQQYYYSPTWTF	M (93.8%)	M (95.3%)
MM025	IgG κ	3-9	2-28	CVQAIRFVF	CMHPVQTAPYIF	M (92.7%)	M (90,8%)
MM026	IgG κ	3-23	3-20	CAQSNVAAAPRGWFDPW	CQQYGNSPGTF	M (96.5%)	M (95%)
MM031	IgG λ	7-4	3-23	CAREYYYNYVRYFDSW	CCSYARDDTFVF	M (85.4%)	M (85.8%)
MM032	IgG λ	2-5	3-1	CVHRRMGQLQDWYFDLW	CQTWDSRTVVF	M (96.2%)	M (90.0%)
MM034	IgG λ	1-69	1-40	CARDTDILVVDVATGFDPW	CQSYDGGRSGSVVF	M (88,5%)	M (87,5%)
MM036	IgG κ	1-f	1-5	CTRSVPSTVHNNWFDPW	CQQYNNFWTF	M (93,8%)	M (97,5%)
MM048	IgA λ	3-21	3-1	CARGGYGDNPYYHYGLDVW	CQAWDSTTVVF	M (92,7%)	M (95.3%)
MM050	IgG κ	3-15	1-5	CATEISSGASVGSVKVLW	CQQYHSYLYTF	M (85,4%)	M (88.1%)

*MM = multiple myeloma, VH = variable heavy chain, VL = variable light chain, HCDR3 = heavy chain complementarity determining region 3, LCDR3 = light chain complementarity determining region 3, Mutational Status = percentage of variable region germline sequence, M = mutated (>98% identity to variable region germline sequence), n.e. = not evaluated.

PCR-based approaches to detect clonotypic B cells may be prone to different kinds of errors as described in the introduction section and may therefore not be able to predict the presence or absence as well as the frequency of these potential myeloma precursor cells. However, in an attempt to compare our patient cohort to the cohorts of other studies, we used a PCR protocol similar to what was used by other investigators to screen PBMC and BMMNC for Ig-positive clonotypic B cells. As previously published work most commonly analyzed IgM-isotype expressing clonotypic cells, a semi-nested PCR approach with HCDR3- and IgM-specific primers was established as illustrated in Figure 1A. Figure 1B shows semi-nested PCR reactions for three exemplary myeloma patients. Whereas patient MM021 and MM025 had clear evidence of clonotypic B cells in peripheral blood and bone marrow (Figure 1B, upper and middle panel), in patient MM036 no specific PCR products could be amplified, suggesting the absence of clonotypic B cells in peripheral blood and bone marrow of this patient (Figure 1B, bottom panel). Of all evaluable patients, 50% exhibited clonotypic cells in the peripheral blood as evidenced by PCR (Table 3). This frequency is in line with data from the literature indicating that 40%–87% of myeloma patients show evidence of clonotypic B cells in the peripheral blood by PCR [15], [17], [18]. In >80% of PCR-positive patients, clonotypic B cells could also be detected in bone marrow (Table 3). However, in this cellular compartment PCR signals were generally weaker, potentially corresponding to the reduced relative frequency of B cells in bone marrow as compared to peripheral blood.

**Table 3:** PCR-detection of clonotypic B cells in myeloma patients.*

MM patient	MM001	MM003	MM008	MM020	MM021	MM022	MM023	MM025	MM026	MM031	MM032	MM034	MM036	MM048	MM050
**PB**	−	n.e.	n.e.	−	+	n.e.	−	+	+	+	−	+	−	+	−
**BM**	n.e.	n.e.	n.e.	n.e.	+	n.e.	−	+	−	+	−	+	−	+	−

*“+” corresponds to detection of clonotypic IgM immunoglobulin rearrangement, “−” corresponds to absence of clonotypic IgM immunoglobulin rearrangement, MM = multiple myeloma, PB = peripheral blood, BM = bone marrow, n.e. = not evaluated due to lack of material.

### Selection of patient-individual ligands specifically binding to myeloma Ig

After having identified patients with PCR-based evidence of clonotypic B cells in the blood and/or bone marrow, we set out to establish a method allowing us to physically label clonotypic cells in an attempt to precisely quantify and eventually characterize this interesting cellular compartment. We chose the patient-individual clonotypic Ig as cellular target for the development of specific ligands mimicking the epitope recognized by this immunoglobulin. This choice was based on two major reasons: First, clonotypic Ig with its highly patient-individual and tumor-specific paratope region is the most reliable clonal marker of this B cell population. Second, clonotypic Ig is expressed on the surface of B cells, rendering it accessible to our labeling strategy.

**Figure 2:**
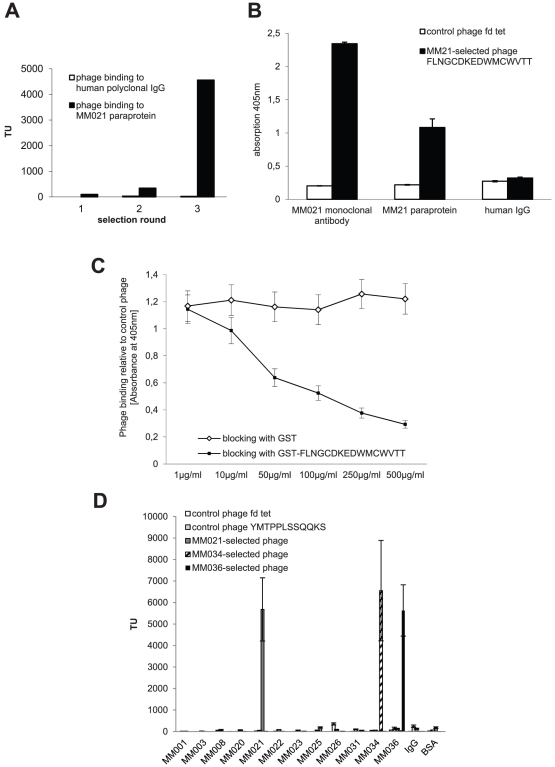
Selection of specific phage-displayed peptide ligands binding to myeloma Ig. **A:** Enrichment of selectively binding phage on MM021 paraprotein over three selection rounds. A X_18_ random peptide phage display library was screened on immobilized MM021 paraprotein. Bound phage were recovered by K91 bacterial infection, amplified overnight, purified and subjected to the next selection round. Negative preselection of the library was performed in selection round two and three. The selected phage were tested for binding to MM021 paraprotein and the control human polyclonal IgG after each selection round. Bacteria transduced by recovered phage were grown on LB plates containing tetracycline to determine the number of transducing units (TU) by colony counting. Ig = immunoglobulin, MM = Multiple Myeloma. **B:** Phage selected on myeloma Ig specifically bind the antibody on which they were selected. Examplarily, binding of paraprotein MM021-selected phage FLNGCDKEDWMCWVTT and control phage fd tet to MM021 monoclonal antibody, MM021 paraprotein and control human IgG is shown. Bound phage were quantified by Enzyme-linked Immunosorbent Assay (ELISA). Data are shown as means from triplicate experiments (± SEM). **C:** The protein GST-FLNGCDKEDWMCWVTT blocks binding of phage FLNGCDKEDWMCWVTT to MM021 paraprotein. MM021-selected phage FLNGCDKEDWMCWVTT was incubated on MM021 paraprotein in the presence of increasing amounts of GST-FLNGCDKEDWMCWVTT or GST alone. Bound phage were quantified as in B. Data are shown as relative values compared to binding of a control phage (means from triplicate experiments ± SEM). GST = glutathione S-transferase. **D:** Phage selected on myeloma Ig specifically bind their target and do not cross-react with other Igs. Binding of phage clones selected on the myeloma Ig of patients MM021, MM034 and MM036 as well as random control phage YMTPPLSSQQKS and fd tet to different myeloma paraproteins and recombinantly expressed Fab fragments (MM001, MM003, MM008, MM020) as well as control polyclonal IgG and Bovine Serum Albumin is shown. Bound phage were quantified as numbers of transducing units (TU) based on bacterial infection (means from triplicate platings ± SEM).

We used combinatorial phage display peptide library screenings selecting epitope-mimicking ligands which specifically bind to myeloma Ig. Peptides were selected on all myeloma Igs, irrespective of PCR-based evidence for clonotypic B cells. For the first phage library selections, myeloma Ig was recombinantly expressed as IgG-Fab in a prokaryotic system (selections on MM001, MM003, MM008 and MM020). As this recombinant approach is very labor-intensive, we also evaluated naturally occurring IgG or IgA paraproteins purified from serum as a source of target Ig. Paraproteins were purified by protein-A (IgG) or jacalin (IgA) chromatography and purity was assessed by coomassie staining of electrophoretically separated eluted Ig fractions (data not shown). Given that all patients had highly elevated Ig serum levels at the time of sample collection, contamination by non-paraprotein IgG or IgA was deemed negligible. Indeed, in most of the cases, binding phage could be enriched over three to four selection rounds on recombinantly expressed Fab as well as on purified paraprotein (exemplarily shown in Figure 2A) with at least one out of four random peptide phage libraries with different insert design (X_7_, X_12_, X_18_ and X_4_CX_6_CX_4_ = beta-sheet conformation [BS]). In only three out of 15 myeloma cases selections did not yield binding phage, potentially indicating that none of our peptide designs mimics the natural epitope of these antibodies. Table 4 lists the myeloma Igs and the peptide sequences selected thereon. All of the selected phage, but not random insert control phage, bound specifically to the myeloma Ig on which they were selected and not to control Igs, exemplarily shown in Figure 2B for MM021-selected phage FLNGCDKEDWMCWVTT binding to MM021 paraprotein. Phage FLNGCDKEDWMCWVTT, selected on the paraprotein of patient MM021, also bound to recombinantly expressed MM021 monoclonal antibody, providing proof of concept that selections on purified paraprotein yield phage clones selectively binding to the clonotypic Ig and not to potentially contaminating normal serum Ig (Figure 2B). The higher binding level of this phage to MM021 monoclonal antibody probably reflects the higher degree of purity as compared to MM021 paraprotein. Likewise, phage selected on recombinantly expressed antibodies bound specifically to the respective patient's native paraprotein verifying correct folding of the recombinant antibody used as target for phage selections (data not shown). To confirm that the selected phage displayed peptides bind the respective myeloma Ig independently of other phage components, we exemplarily performed competition assays with a GST-fusion protein containing the phage-derived peptide FLNGCDKEDWMCWVTT which inhibited binding of phage FLNGCDKEDWMCWVTT to MM021 paraprotein in a concentration-dependent manner (Figure 2C). To evaluate our phage clones for cross-reactivity with different myeloma Igs, we performed extensive binding assays as shown for three exemplary phage clones selected on MM021, MM034 and MM036 as well as respective control phage (Figure 2D). These assays suggest that phage clones selected on myeloma Ig bind their respective target in a highly specific manner and do not cross-react with other myeloma Igs or unrelated proteins.

**Table 4:** Phage displayed peptide sequences binding to myeloma immunoglobulins.*

MM	Lib. X_7_	Lib. X_12_	Lib. X_18_	Lib. BS
001†		YPNYEKWSRWPF	GLPIASPEKYTFWPWKLW	
		SNMTLKELMWPF	TFVGAYRTQWLEQGRFPH	
003†			VWYAWMEDLDHAPEFWKI	
008†			RTPIPKPAWSWRSLAYNS	
			PQDFYRSRFWDAHWMYWQ	
			PRDYRSLLYGFHDLTTKV	
020†		YKTPYHMWQEWV	WYYPDEEAYENFYELWSR	
			AQHNFIETSYDMFQQFRF	
021‡			DRDIYQLSNLVEYLA	FLNGCDKEDWMCWVTT
			GSGYSSNNLVAQKPKATL	
022‡	TDPWSKS		WHICLDERAMCQIKELRV	KSYGCKEERAHCYGQV
	WAPFSLL		QDQYTDCSIKGEHTMVCG	IRMQCREERSSCSMIS
				GTWVCRDERATCRSAS
				SKVVCYDERCTCGRNA
				MSGRCLEHRATCYPWN
023‡	VIGPSSL		TQARLDWVNDEYAPSLR	PELRCLEWIHICYAIG
	YFSPSFH		GILLRSFQEFWLNEEYMY	GRNICLLGDHQCAQVN
			RWENDEFWGMISLKEAML	
			YRWLMRVGIQDRHHQEMG	
025‡			PDLSSQRYIIEGSGQKPQ	SLSPCQVWDAGCQSAG
			PILQYPLVTGTGTKEGIH	REEVCMETVPICDLYF
			PLGQSGRDKAESKTKGWT	GKHLCRPWGLACVAMQ
			PKILSGAGNKVEGASHGA	
			GRAGEKMISGDGNKIHES	
			RDQGYRAPYHVGLGARSG	
026‡				PEQYCCRSRWSCQICH
				SREVVWWDGLSLVRSR
				LLTPCVTRWGNCMQGL
				TPSHCPSIRARCQATI
				HPYLCAMRSDRCAMTV
				AQFLCSDLGRVCTRVS
031‡		SEAIQKNSGNEQ		
		FGQQKTSGNEEG		
		NGLLHQKNAGNE		
		ALHKTSGNEMDI		
		AGHPKDSGNEWA		
034‡		ADSSKRCNFNQC		
		YNGTPSTVHLLP		
036‡		EPYTPHLGQALW		
		IXXRGLPTDMXG		
		GFESSGKYLRKQ		
		VDFPNAWKWWTE		
		SSAGPEQPRLMW		
		YPELPRWFPPNY		
		VPQLPRYYSVSS		
		QIPGWLRTGLEA		

*Libraries with 7-mer linear (X7), 12-mer linear (X12), 18-mer linear (X18), and 14-mer constrained beta sheet (BS) inserts were selected on myeloma Ig of patients MM001–MM036. The table lists the peptide insert sequences (single letter amino acid code) of phage clones for which specific binding to the respective Fab fragment and/or paraprotein was validated in single clone binding assays.

† phage selection on monoclonal Fab fragment.

‡ phage selection on paraprotein.

### Immunofluorescence imaging of surface Ig-positive clonotypic myeloma B cells

**Figure 3:**
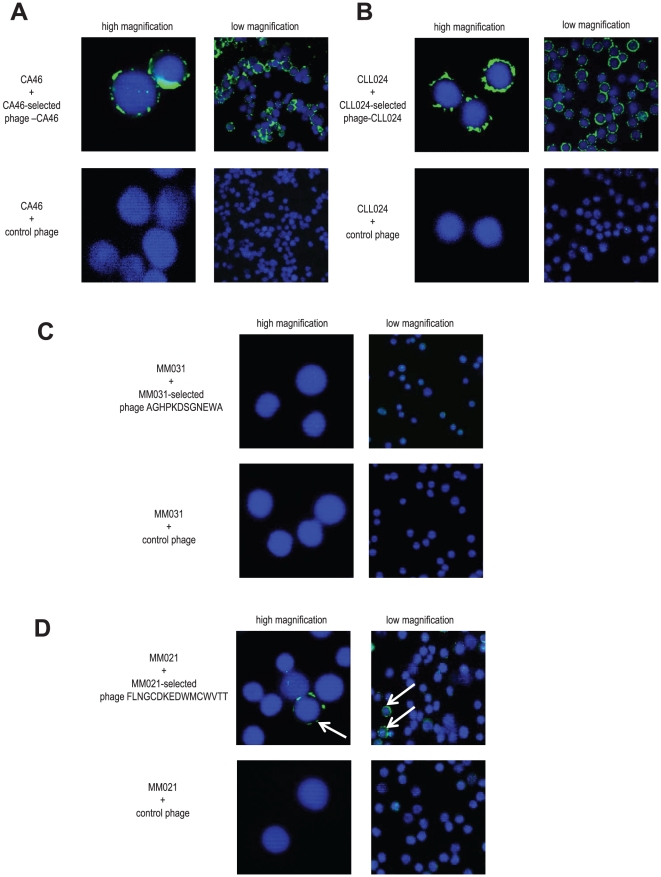
Detection of surface Ig-positive clonotypic B cells by immunofluorescence using patient-individual ligands. **A and B:** Ig-selected phage bind to surface Ig-positive parental B cells as visualized by immunofluorescence. As proof of principal an immunofluorescence staining protocol was set up using two different monoclonal B cell systems as positive controls as described in the Methods and Results sections. Phage-CA46 selected on the Ig of the Burkitt lymphoma cell line CA46 (panel A, upper row) as well as control phage (panel A, bottom row) were used to stain CA46 cells. Phage-CLL024 selected on the Ig of CLL patient 024 (panel B, upper row) as well as control phage (panel B, bottom row) were used to stain primary CLL024 cells. Phage were visualized by immunofluorescence (FITC; green). Nuclei were stained with DAPI (blue). Images are shown at low (right panel) and high magnification (left panel). Images were obtained by confocal microscopy (Leika TCS SP2 AOBS; lens 63×) and analyzed using the Leika confocal software. CLL = chronic lymphocytic leukemia. **C:** Staining of MM031 PBMCs with MM031 Ig-selected phage AGHPKDSGNEWA and control phage to detect potential surface Ig-positive clonotypic B cells. Staining and imaging was performed as in A and B. PBMC = peripheral blood mononuclear cell. **D:** Staining of MM021 PBMCs with MM021 Ig-selected phage FLNGCDKEDWMCWVTT and control phage to detect potential surface Ig-positive clonotypic B cells. Staining and imaging was performed as in A and B. The white arrows point to single surface Ig-positive clonotypic B cells from patient MM021.

Next, we developed an immunofluorescence protocol allowing us to stain clonotypic myeloma B cells from peripheral blood and bone marrow. As such cells do certainly not represent the bulk of PBMCs or BMMNCs, but constitute only a B cell fraction of unknown size, we had to establish our staining protocol using purely monoclonal B cell systems as positive controls. We therefore chose the Burkitt's lymphoma cell line CA46 and primary CLL cells from patient 024 (CLL024), both expressing an individual monoclonal surface IgM. As Ig surface density on clonotypic B cells is unknown so far, these two models seemed particularly suited: Whereas the Burkitt's lymphoma cell line represents a model with high Ig surface density, the CLL model is characterized by extremely low Ig surface expression. The CLL patient's as well as the Burkitt's lymphoma's Ig were recombinantly produced as IgG1 antibodies. Phage displayed peptide ligands were selected targeting the paratope region of the respective monoclonal Igs and subjected to specificity testings as described for the selections on myeloma Igs (Ref. [42] and data not shown). Cells were loaded with the respective selectively binding phage (phage-CA46 for CA46 and phage-CLL024 for CLL024) or control phage, washed, sedimented on slides and cell-bound phage were detected by FITC-labeling. Confocal microscopic imaging revealed bright and specific membrane staining for both cellular systems (green “corona” in Figure 3A and B). CA46 cells stained with phage-CLL024, CLL024 cells stained with phage-CA46 and the control burkitt's lymphoma cell line DG-75 stained with both phage did not show a FITC-signal (data not shown). These findings served as proof-of-principle that phage selected on patient- and clone-specific IgG can be used to specifically label the clonotypic surface IgM on parental cells. In a next step, we stained myeloma PBMCs with the respective phage clones and control phage to screen the samples for clonotypic B cells. As expected, none of the myeloma samples without PCR-detectable clonotypic B cells showed any phage-positive cells by immunofluorescence imaging (data not shown). Surprisingly, however, even most of the PCR-positive samples showed no evidence of specifically phage-labeled cells (exemplarily shown for MM031 in Figure 3C). These findings suggested that only a very slim percentage of myeloma PBMCs can be considered to express the clonotypic Ig rearrangement of the malignant PC clone. Only PBMCs from patient MM021 showed sparse specifically labeled cells (Figure 3D, white arrows) when stained with the Ig-specific phage FLNGCDKEDWMCWVTT, whereas the control phage fd tet did not show any staining. To exclude unspecific binding of phage FLNGCDKEDWMCWVTT, unrelated PBMCs of healthy donors were stained with this phage yielding no specific signals (data not shown). However, as this technique does not allow to exactly determine the frequency of such cells, this data was complemented by precise flow cytometric quantification.

### Flow cytometry-based detection and quantification of surface Ig-positive clonotypic myeloma B cells

**Figure 4:**
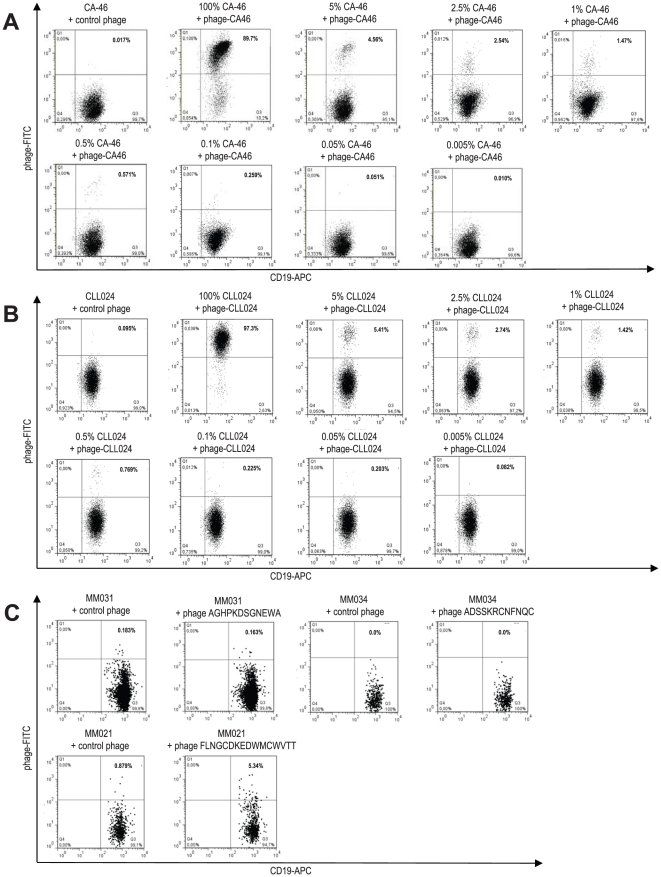
Detection of surface Ig-positive clonotypic B cells by flow cytometry using patient-individual ligands. **A and B:** Ig-selected phage bind to surface Ig-positive parental B cells as detected by flow cytometry. As proof of principal a flow cytometry staining protocol was set up using two different monoclonal B cell systems as positive controls as in the immunofluorescence setting illustrated in Figure 2A and B. Phage-CA46 selected on the Ig of the Burkitt lymphoma cell line CA46 (panel A, second plot) as well as control phage (panel A, first plot) were used to label CA46 cells. Phage-CLL024 selected on the Ig of CLL patient 024 (panel B, second plot) as well as control phage (panel B, first plot) were used to label primary CLL024 cells. Cell-bound phage were stained with fluoresceinisothiocyanate-conjugated secondary detection (denominated “phage-FITC”), B cells were stained with a CD19 allophycocyanin-conjugated antibody (denominated “CD19-APC”). To determine the sensitivity level of the flow cytometry protocol, control phage-stained CA46 cells were spiked with CA46 cells stained with phage-CA46 in a 5%, 2.5%, 1%, 0.5%, 0.1%, 0.05% and 0.005% ratio (panel A, plots 3–7). The same spiking experiment was performed with primary CLL024 cells and the specifically binding phage-CLL024 (panel B, plots 3–7). All measurements recorded 50 000 events on a FACSCalibur flow cytometer (BD) and were analyzed with the FlowJo software (Treestar). **C:** Staining of PBMCs of patients MM031, MM034 and MM021 with phage selected on the respective patients' myeloma Ig (phage AGHPKDSGNEWA, phage ADSSKRCNFNQC and phage FLNGCDKEDWMCWVTT) as well as with control phage to detect potential surface Ig-positive clonotypic B cells. Flow cytometry measurements were performed as in A and B. PBMC = peripheral blood mononuclear cell.

As for the immunofluorescence experiments, CA46 and CLL024 cells were used as positive controls to establish an appropriate flow cytometric protocol to screen the blood and bone marrow of myeloma patients for clonotypic B cells. The two-color flow cytometry staining protocol could be adopted from the immunofluorescence experiments with minor modifications. Cell-bound phage were stained with fluoresceinisothiocyanate-conjugated secondary detection (denominated as “phage-FITC”), B cells were stained with a CD19 allophycocyanin-conjugated antibody (“CD19-APC”). Under these conditions, CA46 and CLL024 cells stained FITC-positive when loaded with their respective specific phage clones (phage-CA46 for CA46 and phage-CLL024 for CLL024) whereas the respective control phage did not show any staining (Figure 4A and B, first two panels). CA46 cells stained with phage-CLL024 and CLL024 cells stained with phage-CA46 did not show a FITC-signal (data not shown). To determine the sensitivity level of this flow cytometry protocol, control phage-stained CA46 cells were then spiked with decreasing amounts of CA46 cells stained with the specifically binding phage-CA46 in a 5%, 2.5%, 1%, 0.5%, 0.1%, 0.05% and 0.005% ratio (Figure 4A). The same spiking experiment was carried out for CLL024 with the specifically binding phage-CLL024 (Figure 4B). In both spiking experiments stained cells could be clearly and repeatedly detected down to ratios of 0.1–0.05%, corresponding to a sensitivity level of this flow cytometry protocol of around 10^−3^.

In further experiments, PBMCs and BMMNCs of myeloma patients as well as healthy donor control PBMCs were stained with specific and control phage for detection of clonotypic B cells in the myeloma samples. All phage used in these experiments were initially tested for unspecific B cell binding on healthy donor control PBMCs and on a number of unrelated myeloma PBMCs. None of the phage clones showed preferential binding as compared to control phage under these control conditions (data not shown). The first two rows of Figure 4C show representative flow cytometric plots of patients MM031 and MM034 after B cell gating. Like these two exemplary patients, most other patients' PBMCs and all BMMNCs were negative for clonotypic B cells by flow cytometry. This further corroborates the immunofluorescence imaging data, indicating that the absolute number as well as the relative percentage of clonotypic B cells may be very low in the majority of myeloma patients. Based on the previously established sensitivity level of this assay, the frequency of clonotypic B cells is less than one clonotypic B cell per 1000 PBMCs in most myeloma patients. Only PBMCs of patient MM021 exhibited detectable amounts of clonotypic B cells by flow cytometry in line with the immunofluorescence data described above (Figure 4C, bottom row). These cells accounted for about 0.15% of PBMCs and 5% of B cells in this patient. In view of the capacity of our assay to detect even minute fractions of idiotype expressing cells by the epitope-mimicking phage, these data show that the actual number of clonotypic cells is exceedingly low (presumably below the assay's detection limit of 10^−3^ cells in the majority of samples, even if PCR suggests their presence).

## Discussion

Clonotypic B cells and their biological significance in multiple myeloma are controversially discussed. While many groups have reproducibly identified Ig-positive B cells expressing the same clonal Ig rearrangement as the malignant PC in about 40–87% of myeloma patients, their proportion relative to normal PBMCs and their potential role in the initiation, relapse and progression of multiple myeloma remains largely unclear [15], [17], [18]. A major limitation to solve this controvery has been the impossibility to morphologically identify or directly isolate these clonotypic cells for further functional analyses to determine their biological role.

Here, we present a technique to phenotypically detect, quantify and isolate clonotypic myeloma B cells by flow cytometry by means of epitope-mimicking ligands highly specific for binding to myeloma-idiotypic surface immunoglobulin, independently of Ig isotype. Our cohort comprised 15 patients with confirmed multiple myeloma at different disease stages. Fifty percent of informative patients showed evidence of clonotypic B cells in peripheral blood and/or bone marrow by semi-nested PCR using HCDR3-specific patient-individual primers, in line with published data. Surprisingly, in most patients with PCR-positivity for clonotypic B cells, the number and relative proportion of such cells was below the sensitivity level of our flow cytometry protocol. This sensitivity level had precisely been determined by spiking experiments using two different monoclonal B cell systems with different Ig receptor density (Burkitt's lymphoma CA46 and CLL024) to reliably range around 10^−3^. This indicates that in the majority of myeloma patients, less than one out of 1000 PBMCs or BMMNCs corresponds to a clonotypic B cell. Only one patient of our cohort had detectable amounts of clonotypic B cells by flow cytometry and immunofluorescence imaging, accounting for about 0.15% of PBMCs and 5% of B cells. These findings clearly contradict the estimates of 0.24%–25% of PBMCs and up to two thirds of B cells suggested to be clonotypic B cells by previously published limited dilution PCR assays [15], [16], [17]. Although for the time being there is no definitive explanation for this unexpected discrepancy, the qualitative detection of clonotypic B cells by semi-nested PCR could be flawed for example by unspecific annealing of the CDR3-primer. Such unspecific annealing may seem implausible at first glance as this immunoglobulin region represents the most variable part of the antibody. However, the diversity in this region is created in part by reading frame shifts, which result in different amino acid sequences, whereas the DNA sequence can be similar or even identical in different antibody clones. This would allow for primer annealing in antibody clones with different CDR3 amino acid sequences giving rise to false positive PCR results and potentially to an overestimation of the frequency of clonotypic B cells by PCR-based approaches.

From the first description of clonotypic B cell Ig rearrangements in 1993 (Ref. [11]) until today, there has been much debate about the biological significance of clonotypic B cells in myeloma as outlined in the introduction section of this manuscript. Animal models ascribing proliferative potential exclusively to clonotypic B cells and the estimated frequency of such cells in remission and relapse have supported the hypothesis that clonotypic B cells may continuously replenish the tumor compartment as “feeder” cells [14], [15], [16], [22], [23], [24], [26], [27], [28], [29]. On the other hand, alternative animal models and recent genetic analyzes of clonal hierarchies as well as class switch recombination events in the B cell lineage of multiple myeloma patients challenge the myeloma stem cell hypothesis [30], [31], [33], [35], [37], [38], [39]. The prominent role assumed by clonotypic B cells over the past years of myeloma research has probably – amongst other factors – to do with the surprisingly high percentages of such cells found by PCR-based quantification. If clonotypic B cells on average constituted about two thirds of all B cells in myeloma patients and if they remained constant or even increased over the course of the disease, they would have to have a survival advantage compared to normal B cells. Either this survival advantage would reflect a (pre-) malignant state of such B cells or – although less likely – clonotypic B cell survival and proliferation would have to be driven by some external signal, e.g. a persisting, non-eliminated antigen that had selected the B cell clone in the first place. Both hypotheses, particularly the first one, would be highly compatible with a “feeder” function of clonotypic B cells. On the other hand, much lower levels of clonotypic B cells, as suggested by our study, do not necessarily contradict these hypotheses and especially do not exclude a causal role of these cells as progenitor cells in disease progression. It should be pointed out, however, that only a limited percentage of myeloma patients do have PCR-based evidence of B cells clonally related to the malignant plasma cell clone (50% in our study) and even in PCR-positive patients, clonotypic B cells seem to be a very rare event according to our data. Therefore, it seems rather implausible that a clonotypic B cell compartment is an indispensable “feeder” of the malignant PC compartment and an essential prerequisite of myeloma maintenance and progression. Given the apparent rarity of clonotypic B cells in myeloma, it is conceivable that these cells are non-malignant remnants, which may have initially given rise to the malignant plasma cell clone, but which do not have any significance in tumor maintenance. Taking into account the long term persistence of such B cell clones over the course of the disease, they may in fact resemble long-lived memory B cells with some sort of self-renewing properties.

Detection of clonotypic B cells in this study as well as in others has been limited so far to the peripheral blood and bone marrow. This choice has certainly not been made merely for scientific interest, but primarily because these cellular compartments are easily accessible in myeloma patients. However, to get a more comprehensive picture about clonotypic B cells in multiple myeloma, it may be of interest to study other lymphatic tissues, such as spleen or lymph nodes to exclude that these organs possibly serve as niches harboring clonotypic B cells. Our established flow cytometry protocol may serve as a tool to quantitate clonogenic B cells also from different lymphoid organs.

Taken together, this is the first report on phenotypic detection and quantification of clonotypic myeloma B cells using highly patient- and clone-specific ligands as tracers for this rare B cell population. Our new approach not only serves to precisely quantify this B cell population in a complementary way to previous quantification attempts. More importantly, by phenotypic detection and isolation of pure populations of clonotypic B cells, it may pave the way to experimentally address some of the apparent inconsistencies and controversies regarding the clinical role of clonotypic B cells in multiple myeloma in the future.

## Supporting Information

Table S1 **Primer design for (semi-nested) PCRs.**
(DOC)
