# Supplementary material for: Phenotypic Detection of Clonotypic B Cells in Multiple Myeloma by Specific Immunoglobulin Ligands Reveals their Rarity in Multiple Myeloma
Source: PLoS One. 2012 Feb 22;7(2):e31998. doi: 10.1371/journal.pone.0031998 (PMC3285203; doi:10.1371/journal.pone.0031998)
Supplement: Table S1 — Primer design for (semi-nested) PCRs. (DOC) [file pone.0031998.s001.doc]

*Table S1: Primer design for (semi-nested) PCRs.**

* VH = variable heavy chain, VL = variable light chain, HCDR3 = heavy chain complementarity determining region 3. HCDR3 primers marked with two asteriskes (**) were designed as reverse primers as opposed to all other HCDR3 (forward) primers.The semi-nested PCR in these cases was carried out in a slightly modified way, using family-specific forward leader primers for the secondary amplification step.
